# Prevalence of Reversed Genome Organizations for Viruses in the Family *Iflaviridae*, Order *Picornavirales*

**DOI:** 10.1128/spectrum.04738-22

**Published:** 2023-05-01

**Authors:** Yan Zhang, Zhuang-Xin Ye, Xiao-Xiao Feng, Zhong-Tian Xu, Jian-Ping Chen, Chuan-Xi Zhang, Jun-Min Li

**Affiliations:** a State Key Laboratory for Managing Biotic and Chemical Threats to the Quality and Safety of Agro-products, Institute of Plant Virology, Ningbo University, Ningbo, China; b Key Laboratory of Biotechnology in Plant Protection of MARA and Zhejiang Province, Institute of Plant Virology, Ningbo University, Ningbo, China; c College of Plant Protection, Northwest Agriculture and Forestry University, Yangling, Shaanxi, China; d Agricultural Experiment Station, Zhejiang University, Hangzhou, China; Instituto de Histología y Embriología de Mendoza (IHEM)

**Keywords:** picornavirus, iflavirus, virus taxonomy, reversed genome organization

## Abstract

Viruses in the order *Picornavirales* possess a positive-strand RNA genome that encodes structural proteins (SPs) and nonstructural proteins (NSPs). According to the recent report of the International Committee on Taxonomy of Viruses (ICTV), there are 8 families in *Picornavirales*, and monopartite picornaviruses in each family exhibit distinct types of genome organizations with rearranged genes coding for SPs and NSPs, namely, TypeI (5′-SPs-NSPs-3′) and TypeII (5′-NSPs-SPs-3′). In the present study, 2 iflaviruses with the 2 genome types were unexpectedly identified in a damselfly host species, suggesting that these 2 genome types coexisted in the same host species, and the families of order *Picornavirales* might be more complex than previously thought. The consequent systematic homologous screening with all the publicly available picornaviruses successfully revealed a considerable number of candidates rearranged genome types of picornaviruses in various families of *Picornavirales*. Subsequently, phylogenetic trees were reconstructed based on RNA dependent RNA polymerase and coat protein, which evidently confirmed the prevalence of the 10 typeII iflaviruses in the *Iflaviridae* family. This suggests that genome types may not be relevant to viral taxonomy in this family. However, candidate picornaviruses with reversed genome types in the *Secoviridae* and *Dicistroviridae* families require further investigation. All in all, as the number of newly discovered viruses increases, more viruses with non-canonical genome arrangements will be uncovered, which can expand our current knowledge on the genome complexity and evolution of picornaviruses.

**IMPORTANCE** Monopartite viruses in the order *Picornavirales* exhibit distinct genome arrangement of nonstructural proteins and structural proteins for each of the 8 families. Recent studies indicated that at least 4 ifla-like viruses possessed reversed genome organization in the family *Iflaviridae*, raising the possibility that this phenomenon may commonly present in different families of picornaviruses. Since we discovered 2 iflaviruses with exchanged structural and nonstructural proteins simultaneously in the damselfly, a systematic screening was subsequently performed for all of the current available picornaviruses (1,543 candidates). The results revealed 10 picornaviruses with reversed genome organization in the family *Iflaviridae*, implying that this phenomenon might prevalence in the order *Picornavirales*. These results will contribute to a better understanding for the future study on the genome complexity and taxonomy of picornaviruses.

## INTRODUCTION

RNA viruses are notable for their high variability in genome structures, rapid evolution, and adaptation (1). As reported by the International Committee on Taxonomy of Viruses (ICTV), the taxonomy of RNA viruses generally depends on viral genetic relatedness, genome organization, virion characteristics, and biological properties such as host range (2). The genomes of RNA viruses can encode a limited number of proteins, among which RNA dependent RNA polymerase (RdRp) is essential for viral RNA replication and is the key protein used for the taxonomy of different RNA viruses (3). It is believed that a group of evolutionarily related viruses tend to have genetic similarity, homogeneous distinct genome organizations, analogous expression features, and share related hosts (4).

As one of the most important viral orders, *Picornavirales* contains viruses with a monopartite or bipartite positive-strand RNA genome ranging from 7,000 to 12,500 nucleotides (nt) in length (3). The viral genome is post-translationally processed by the viral protease into various precursor and mature proteins, including structural proteins (SPs) and nonstructural proteins (NSPs). Of them, SPs usually contain 3 rhinovirus-like (rhv-like) capsid domains (or a cricket paralysis virus-like [CRPV-like] capsid domain for some picornaviruses), while NSPs consist of a viral helicase (Hel) domain, a 3-chymotrypsin-like protease (Pro) domain and the RdRp domain (5). According to the latest version of MSL37 (Master Species List, https://ictv.global/msl), there are 8 families in the order *Picornavirales* with highly variable genome organizations. The majority of the viruses in *Picornavirales* are generally unsegmented with the monopartite genome (except for several genera of the family *Secoviridae*). For monopartite viruses in the families of *Iflaviridae* (6), *Picornaviridae* (5), *Polycipiviridae* (7), and *Secoviridae* (genera of *Sequivirus* and *Waikavirus*) (8), SPs are distributed in the N-terminal region of the polyprotein, whereas NSPs in the C-terminal region (5′-SPs-NSPs-3′, referred to as typeI hereafter). Conversely, reversed genome organization with the order 5′-NSPs-SPs-3′ (referred to as typeII hereafter) can be observed in the families of *Caliciviridae* (9), *Dicistroviridae* (10), *Marnaviridae* (11), and *Solinviviridae* (12). According to a recent ICTV online report (https://ictv.global/report), monopartite picornaviruses in each family of the order *Picornavirales* exhibit distinct types of genome organizations (typeI or typeII).

The family *Iflaviridae* possesses a positive-stranded RNA genome (mono-cistronic genome) that is approximately 9 to 11 kb in length, with the typeI genome organization. Currently, there is only 1 genus *Iflavirus* in the family *Iflaviridae*, and iflaviruses are identified exclusively from arthropods, primarily from insects (6). Although the genome arrangement of all ICTV accepted viruses in the family *Iflaviridae* is typeI (16 species), recent studies have provided convincing evidence that the typeII genome organization can be unexpectedly presented in at least another 4 ifla-like viruses, including *Diaphorina citri* picorna-like virus (DcPLV) (13), *Riptortus pedestris* virus-2 (RiPV2) (14), *Pectinophora gossypiella* virus 4 (PecgV4) (15), and *Bactericera cockerelli* picorna-like virus (BcPLV) (16). Phylogenetic analysis shows that these 4 viruses may belong to the family *Iflaviridae*, raising the possibility that the 2 types of genome organizations may simultaneously exist in this family. It has also been proposed to establish a new genus “*Psylloidivirus*” based on the typeII genome organization of viruses discovered in *Iflaviridae* (16). Nevertheless, considering the limited number of iflaviruses (16 species) officially accepted by ICTV, while a substantial number of iflaviruses are currently available in the public database, whether there are more iflaviruses with typeII genome organization is still unexplored and their authentic taxonomical statutes should be further investigated. Moreover, it is also interesting to explore the complexity of genome arrangement in other families of the order *Picornavirales*.

In this study, 2 iflaviruses with exchanged SPs and NSPs domains were discovered in the same insect species. Moreover, systematic screening of the currently available picornaviruses successfully identified multiple unexpected genome complexities of viruses in the families of *Iflaviridae*, *Secoviridae*, and *Dicistroviridae*. This study will facilitate a better understanding for the viral genome diversity in the order *Picornavirales*.

## RESULTS

### Two iflaviruses with different genome organization types identified in damselfly.

**FIG 1:**
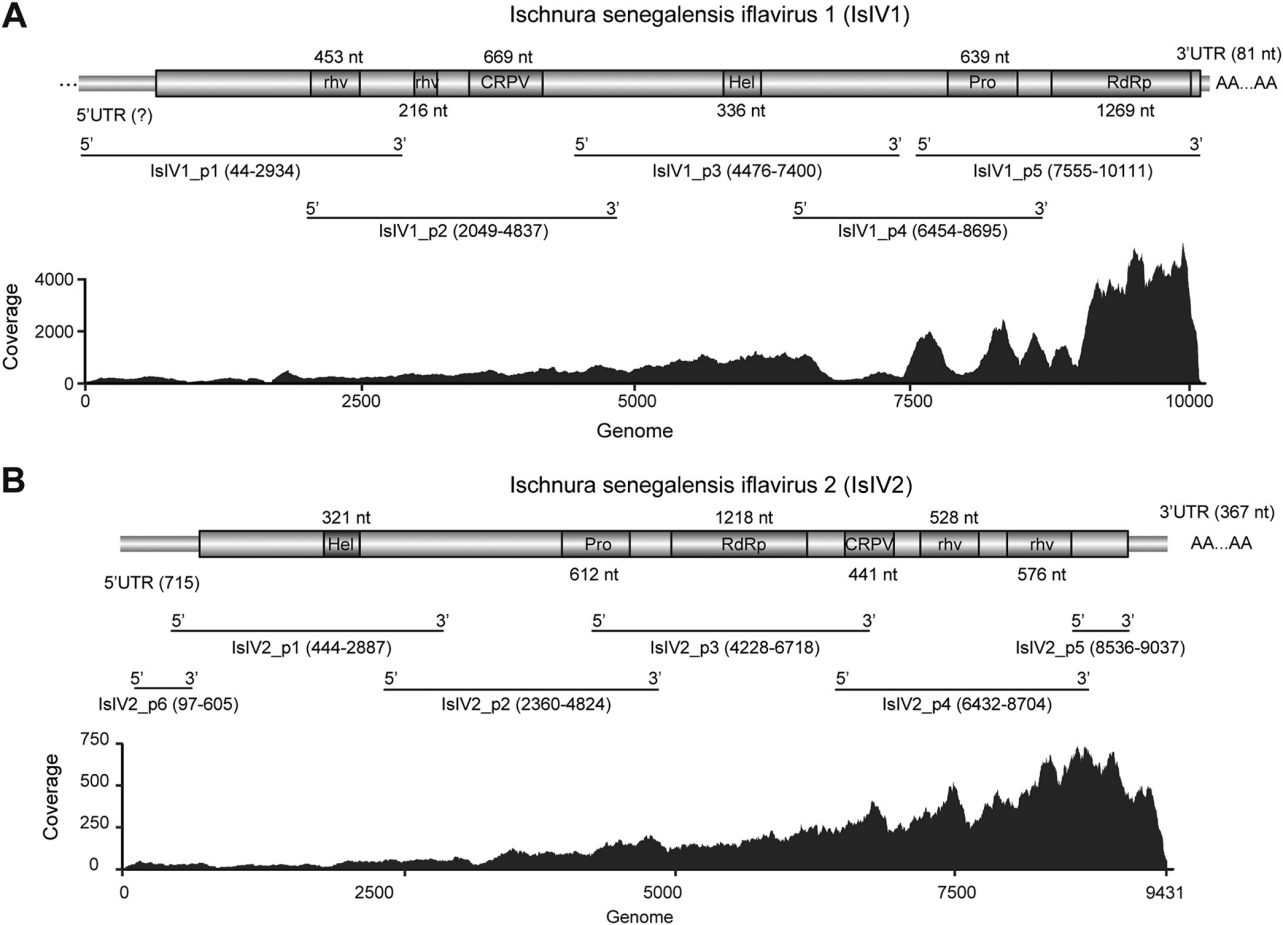
Genome structure and read coverage of Ischnura senegalensis iflavirus 1 (A) and Ischnura senegalensis iflavirus 2 (B). Positions of the designed primer sets were shown beneath the genome structures of each virus. Abbreviations: rhv, picornaviruslike capsid domain; CRPV, cricket paralysis virus capsid superfamily domain; Hel, viral helicase domain; Pro, 3-chymotrypsin-like protease; RdRp, RNA dependent RNA polymerase domain.

A total of 92,873 contigs were obtained from the *de novo* assembled clean reads, and the species of the damselfly is *Ischnura senegalensis* (family Coenagrionidae) with the COI sequence identical to the previously reported one (Accession: NC_060418.1). Through homology search for the viral sequences, 2 contigs representing potential iflaviruses with nearly complete genomes were identified and named Ischnura senegalensis Iflavirus 1 (IsIV1) and Ischnura senegalensis Iflavirus 2 (IsIV2), respectively. These 2 viral contigs were further verified by RT-PCR, followed by Sanger sequencing. It should be noted that the products of one primer set (IsIV1_p3 and IsIV2_p3) were designed to span the junctions of SPs and NSPs for the 2 viruses in order to eliminate false positives (such as recombination, misassembled contigs, and others) as indicated in Fig. 1. In addition, 3′ termini of IsIV1 and both termini of IsIV2 (complete genome) were successfully determined. The genome sequences of IsIV1 (10,191 nt) and IsIV2 (9,431 nt) were submitted to GenBank with the accession numbers OP548101 and OP548102, respectively (File S1). Both IsIV1 and IsIV2 are predicted to contain only 1 open reading frame (ORF), which encodes the corresponding polyproteins with the amino acid (aa) length of 3,136 and 2,781, respectively. Conserved domain analysis showed that IsIV1 exhibits typical typeI genome arrangement with SPs (rhv, rhv, and CRPV) in the N-terminus and NSPs (Hel, Pro, and RdRp) in the C-terminus (Fig. 1A). For IsIV2, it is noteworthy that the viral genome organization is typeII, with NSPs (Hel, Pro, and RdRp) in the N-terminus whereas SPs (CRPV, rhv, and rhv) in the C-terminus (Fig. 1B), suggesting that these genome organization types of iflaviruses can exist in the same host species. Moreover, the reads coverage rates were relatively high in the 3′-terminus of genomic RNAs, even though the conserved domains in the 3′ termini are totally different for IsIV1 and IsIV2 (Fig. 1). According to a homology search against NCBI NR database, SPs region (CP) of IsIV1 shares the percentages of 96.73%, 64.81%, and 30.16% to Hangzhou *lschnura senegalensis* iflavirus 1 (IsIV1-Hz, accession: UHK03231.1), Sanya *Ischnura senegalensis* iflavirus 1 (IsIV1-Sy, accession: UHM27540.1), and *Halyomorpha halys* ifla-like virus 1 (accession: URQ09133.1), respectively. Based on the demarcation criteria for a species in the family *Iflaviridae* (sequence identity of CP protein above 90%) (https://ictv.global/report/chapter/iflaviridae/iflaviridae), IsIV1 and IsIV1-Hz are different isolates of the same species, whereas IsIV1-Sy is a different species to IsIV1, although these 3 viruses share the same host insect (*I. senegalensis*). Similarly, a homology search with CP region of IsIV2 showed that the most closely related virus is *Frankliniella occidentalis* associated iflavirus 1 (accession: QNM37810.1) with the CP identity of 40.56%, indicating that IsIV2 is a novel iflavirus.

### IsIV1 and IsIV2 induce host small interfering RNA-based antiviral pathway.

**FIG 2:**
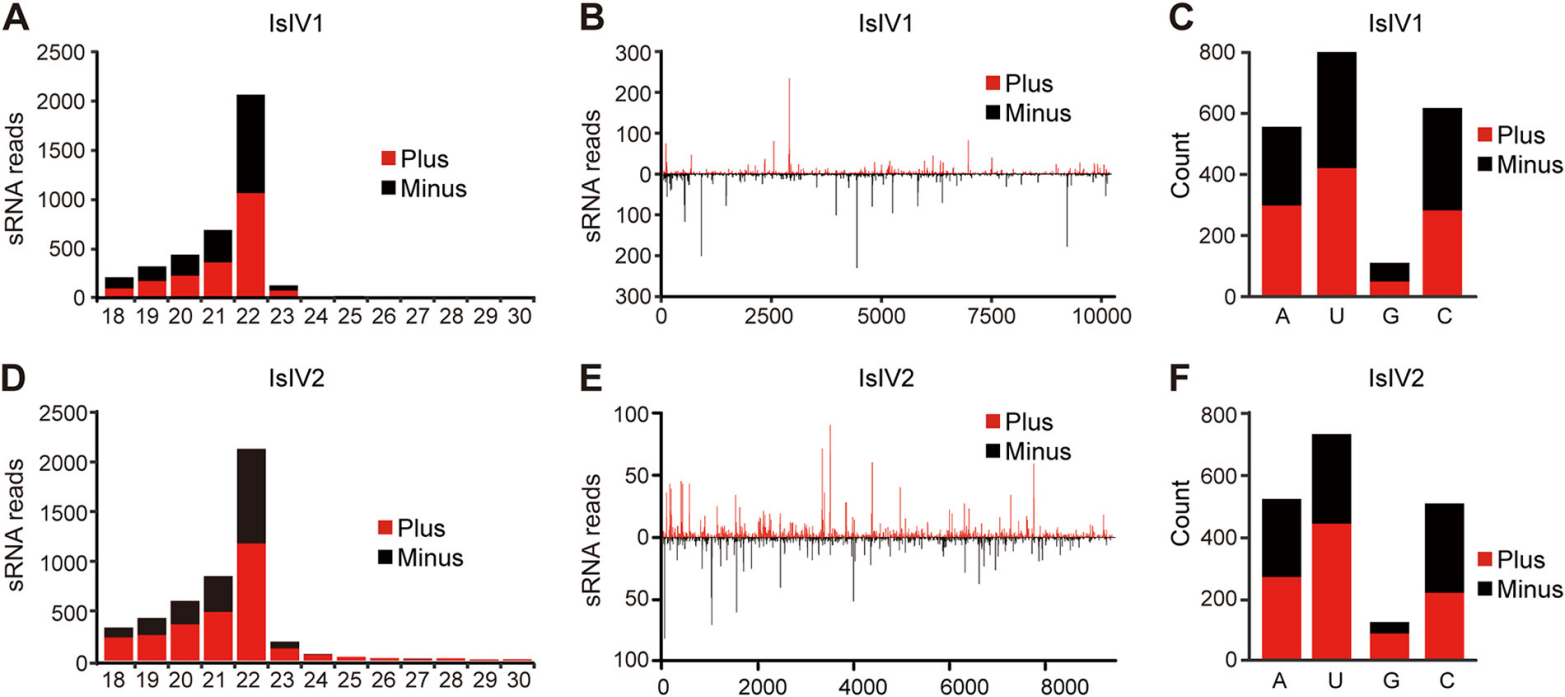
Profiles of virus-derived small interfering RNAs (vsiRNAs) of Ischnura senegalensis iflavirus 1 (IsIV1) and Ischnura senegalensis iflavirus 2 (IsIV2). The size distribution of IsIV1 and IsIV2 derived siRNAs (non-redundant reads) (A) and (D). Distribution of IsIV1 and IsIV2 derived siRNA across the viral genome (B) and (E). 5′ terminal nucleotide preference of siRNAs derived from IsIV1 and IsIV2 (C) and (F). Red represents siRNAs derived from the viral sense genomic strand (Plus), and black represents small RNAs derived from the viral antisense genomic strand (Minus). All the virus-derived siRNAs used for this analysis are redundant (total siRNAs).

Accumulation of vsiRNAs is commonly observed in siRNA-based antiviral immunity in insect hosts infected by exogenous viruses (17). Analysis of sRNAs in *I. senegalensis* showed that a large number of siRNAs (18 to 30 nt) derived from IsIV1 and IsIV2 were identified in the sequenced library. Specifically, a total of 9,446 siRNA reads (3,884 unique) were mapped perfectly to IsIV2, while 11867 were mapped to IsIV1 (3916 unique). These vsiRNAs showed a clear preference of 22 nt in length (accounting for 45.11% and 52.73% of total vsiRNAs in IsIV2 and IsIV1, respectively) and they were equally derived from the plus and minus strands of viral genomic RNAs (Fig. 2A and D). Notably, asymmetric hot spots of vsiRNAs were observed alongside the genomes for both iflaviruses, although their SPs and NSPs regions were interchanged (Fig. 2B and E). Moreover, vsiRNAs of IsIV1 and IsIV2 exhibited a strong A/U bias in the 5′ terminal nucleotides (Fig. 2C and F). As revealed by the representative features of vsiRNAs, the siRNA-based antiviral pathway was involved in the response to the replication of IsIV1 and IsIV2 in *I. senegalensis*.

### Systematic screening of candidate picornaviruses with inverted genome organization in the eight families of the order *Picornavirales*.

All the publicly available picornaviruses were retrieved from a public database, and the preliminary classification of viruses was performed based on their homology to the reference genome of picornaviruses. As a result, the candidate picornaviruses in each family included 441 in *Iflaviridae*, 662 in *Picornaviridae*, 18 in *Polycipiviridae*, 135 in *Secoviridae*, 100 in *Caliciviridae*, 144 in *Dicistroviridae*, 36 in *Marnaviridae*, and 7 in *Solinviviridae*. Genome sequences of candidate picornaviruses are provided in File S2. In accordance with the visualized genome organization of these picornaviruses shown in File S3, along with a preliminary reconstructed phylogenetic analysis for each of the family (excluding of the obviously misclassified picornaviruses), a considerable amount of candidate picornaviruses with the non-canonical genome arrangement were discovered or re-discovered, possibly originating from 3 families. These included 10 viruses (TypeII) in *Iflaviridae*, 1 (TypeII) in *Secoviridae*, and 8 (TypeII-1 and TypeI) in *Dicistroviridae*. Nevertheless, picornaviruses belonging to the other 5 families (*Picornaviridae*, *Polycipiviridae*, *Caliciviridae*, *Marnaviridae*, and *Solinviviridae*) exhibited the same distinct genome structure patterns, consistent with the recent ICTV online report (https://ictv.global/report).

### Candidate picornaviruses with a non-canonical genome arrangement in the viral families of *Iflaviridae*, *Secoviridae*, and *Dicistroviridae*.

**FIG 3:**
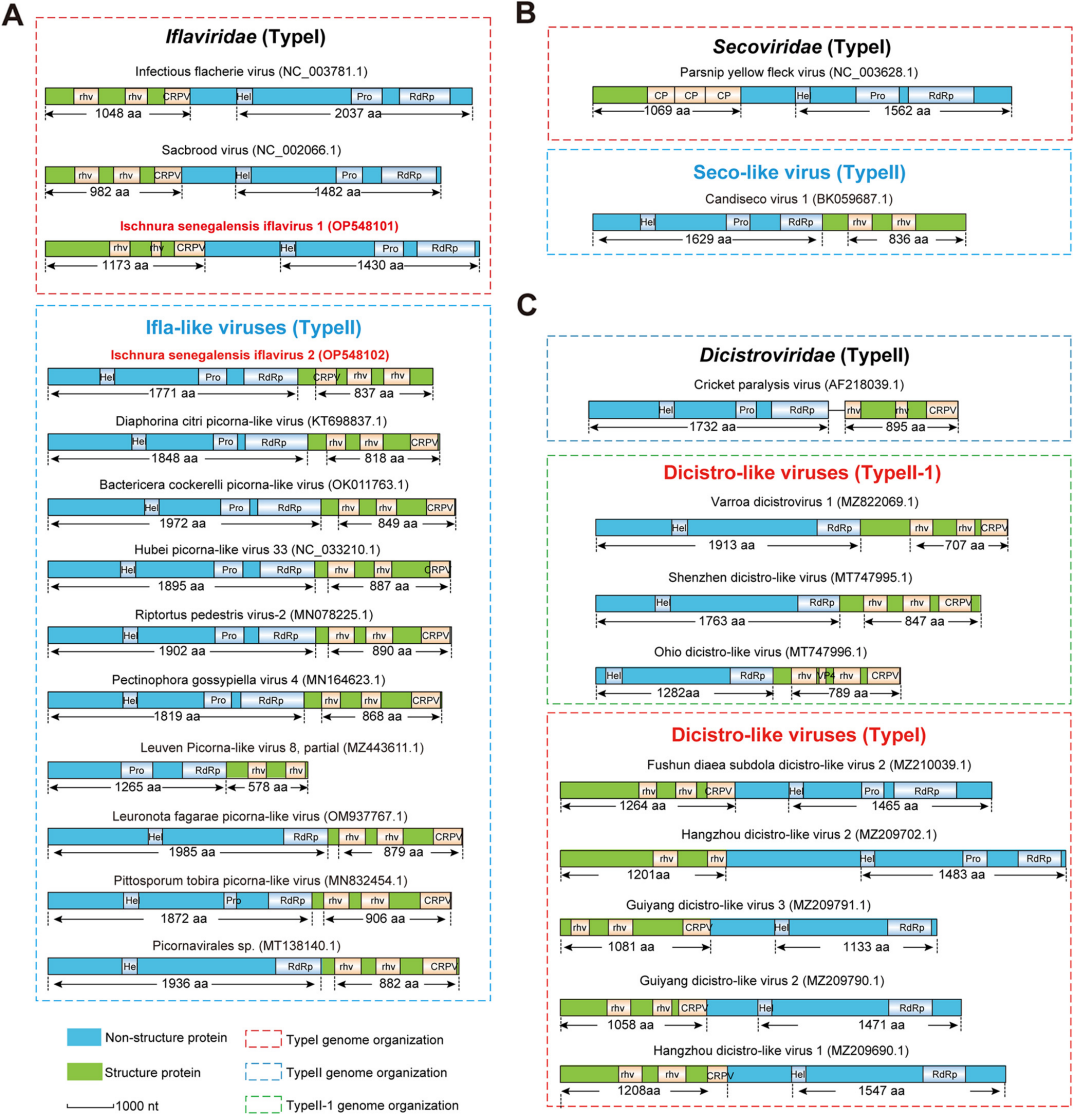
Genome structure analysis of picornaviruses with canonical and non-canonical genome types. (A) Genome organization of iflaviruses with canonical (TypeI) and non-canonical (TypeII) genome types. (B) Genome organization of secoviruses with canonical (TypeI) and non-canonical (TypeII) genome types. (C) Genome organization of dicistroviruses with canonical (TypeII) and non-canonical (TypeI and TypeII-1) genome types. Structural proteins and nonstructural proteins are shown with green and blue box, respectively. The pink, blue, and green boxes (dotted line) represent TypeI, TypeII, and TypeII-1 genome organizations, respectively.

Viruses in the family *Iflaviridae* are officially recognized to have a genome organization of TypeI (https://ictv.global/report/chapter/iflaviridae/iflaviridae), although another 4 iflaviruses (DcPLV, RiPV2, PecgV4, and BcPLV) have been independently reported to possess the genome of TypeII (13–16). *In silico* screening resulted in the successful discovery of another 6 ifla-like viruses with a TypeII genome (including IsIV2 identified in this study), suggesting that the phenomenon of inverted NSPs and SPs might be commonly presented in the family *Iflaviridae* (Fig. 3A).

Viruses belonging to the *Sequivirus* and *Waikavirus* genera (family *Secoviridae*) have a mono-cistronic genome of typeI (8). To date, no virus with a TypeII genome have been reported in these 2 genera. However, our screening results have identified a seco-like virus, Candiseco virus 1 (CDV1, Accession: BK059687.1), with a type II genome (Fig. 3B).

Viruses in the family *Dicistroviridae* possess a monopartite, singe-stranded RNA genome of approximately 8 to 10 kb in size, consisting of 2 non-overlapping ORFs (ORF1 and ORF2) separated by an intergenic untranslated region of about 170 to 530 nt in length (a bi-cistronic genome) (10). Typically, the NSP (ORF1) and SP (ORF2) precursors are encoded by the 5′-proximal and 3′-proximal ORFs, respectively, reflecting the TypeII genome organization. However, *in silico* screening of this family has revealed some candidate dicistroviruses with inverted genome structures of NSP and SP (TypeI) (Fig. 3C). It should be noted that all of the candidate dicistroviruses exhibiting a TypeI genome contain only 1 ORF that encodes a polyprotein (mono-cistronic), similar to iflaviruses, and is totally different compared to the classical bi-cistronic genome structure of the officially reported dicistroviruses (10). Moreover, 3 other candidate dicistroviruses with the conventional typeII genome but with a mono-cistronic structure were also unexpectedly identified (termed as TypeII-1) in this analysis (Fig. 3C).

### Phylogenetic analysis confirmed the prevalence of a reversed genome arrangement in the family *Iflaviridae*.

**FIG 4:**
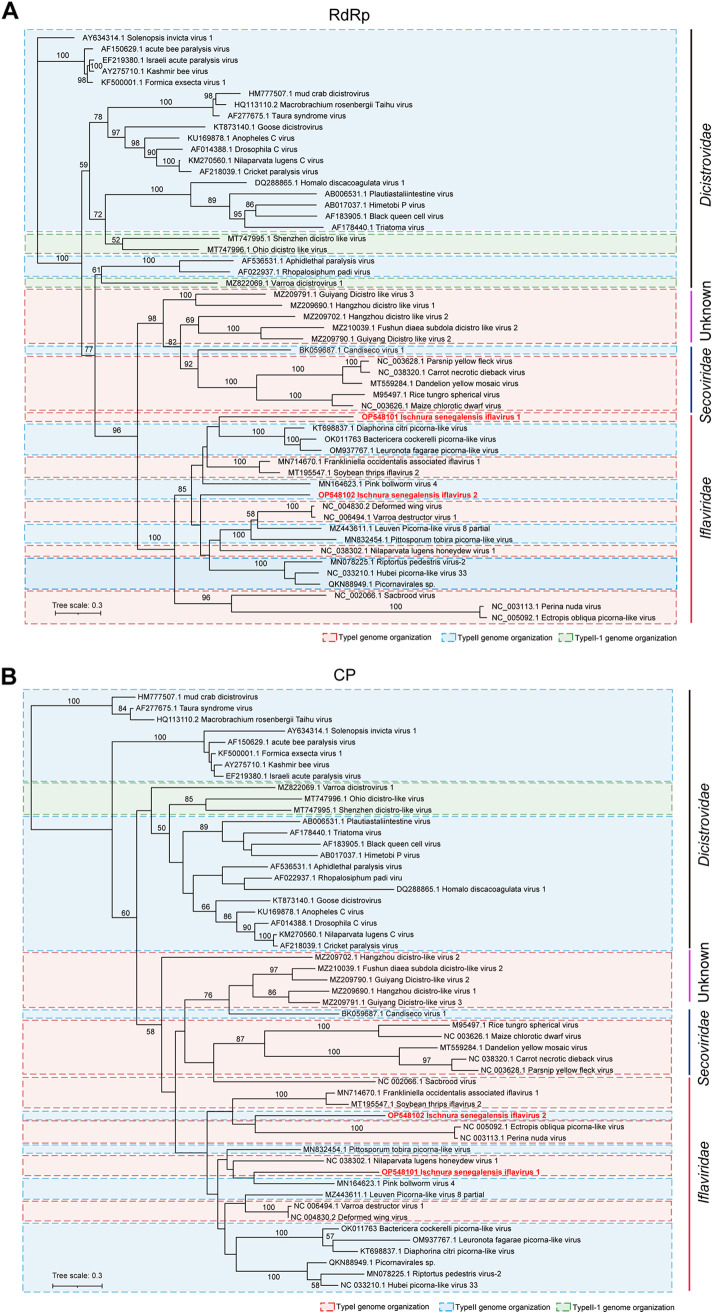
Maximum likelihood phylogenetic tree of picornaviruses in the families of *Iflaviridae*, *Secoviridae*, and *Dicistrovidae* constructed based on the deduced RNA dependent RNA polymerase (A) and coat protein (B) domain. Bootstrap values are shown at each node of the tree. Scale bars represent percent divergence. Two iflaviruses identified in this study are indicated with red bold font. The pink, blue, and green boxes (dotted line) represent TypeI, TypeII, and TypeII-1 genome organizations, respectively.

To confirm the authentic taxonomical status of picornaviruses with non-canonical genome arrangement, phylogenetic trees based on RdRp (Fig. 4A) and CP (Fig. 4B) of representative picornaviruses from these 3 families were constructed. For the picornaviruses in the family *Iflaviridae*, it was evident that the 10 iflaviruses with TypeII genome (rectangle with blue) were scattered within the trees (Fig. 4), thereby confirming the prevalence of the inversed genome organizations in this family (Table 1) and potentially indicating that genome arrangement may be irrelevant for iflavirus taxonomy. It is also noteworthy that IsIV1 and IsIV2 were discovered in the same insect host *I. senegalensis*, indicating that the 2 genome types of iflaviruses can coexist in the same host species.

**TABLE 1:** Novel genome organization type (TypeII) of viruses in the viral family of *Iflaviridae*

Virus names	NCBI accession	Length (bp)	Host species	Host family	Host order
Ischnura senegalensis iflavirus 2	OP548102	9,431	*Ischnura senegalensis*	Coenagrionidae	Odonata
Hubei picorna-like virus 33	NC_033210.1	9,658	N/A	N/A	Odonata
Pectinophora gossypiella virus 4	MN164623.1	9,782	*Pectinophora gossypiella*	Gelechiidae	Lepidoptera
Diaphorina citri picorna-like virus	KT698837.1	8,496	*Diaphorina citri*	Liviidae	Hemiptera
Bactericera cockerelli picorna-like virus	OK011763	9,939	*Bactericera cockerelli*	Triozidae	Hemiptera
Riptortus pedestris virus-2	MN078225.1	9,915	*Riptortus pedestris*	Alydidae	Hemiptera
Pittosporum tobira picorna-like virus	MN832454.1	9,750	*Pittosporum tobira*	Pittosporaceae	Apiales
Leuronota fagarae picorna-like virus	OM937767.1	10,006	*Leuronota fagarae*	Triozidae	Hemiptera
Leuven Picorna-like virus 8, partial	MZ443611.1	5,530	*Vespula vulgaris*	Vespidae	Hymenoptera
Picornavirales sp.	MT138140.1	9,222	*Schoeniclus spodocephala*	Fringillidae	Passeriformes

For the picornaviruses in the family *Secoviridae*, phylogenetic tree based on RdRP showed that CDV1 clustered with other monopartite secoviruses (sequiviruses and waikaviruses) with high bootstrap support (Fig. 4A). However, when the phylogenetic tree was constructed using CP, CDV1 was clearly separated from other secoviruses (Fig. 4B). Given that CDV1 was discovered through large-scale virome data mining (18), and its inconsistent phylogenetic status in the 2 trees, further investigation is needed to confirm CDV1 is a new member of the family *Secoviridae*. Moreover, while currently reported secoviruses are believed to infect plant hosts (or be transmitted by insects), CDV1 was found in a flatworm (*Bdelloura candida*) (8). It is suspected that the host of CDV1 might not be accurately identified or potentially derived from contamination.

The phylogenetic analysis revealed that the 5 dicistro-like viruses with a TypeI genome (indicated by a red rectangle) in the *Dicistroviridae* family were distinctly separated from the dicistroviruses recognized by ICTV in both the RdRP- and CP-based trees (Fig. 4A and B). This suggests that these viruses may not belong to the *Dicistroviridae* family and instead may represent a new taxonomic group within the order *Picornavirales*. Meanwhile, the 3 dicistro-like viruses with TypeII-1 genome (indicated by a green rectangle) were dispersed within the family *Dicistroviridae* with relatively low bootstrap support (Fig. 4). Regarding the classical bi-cistronic genome structure of ICTV-defined dicistroviruses (10), the mono-cistronic genome structure and the uncertainty taxonomically status imply that the identified dicistro-like viruses with TypeI and TypeII-I genomes may not be members of *Dicistroviridae* family and may instead represent new taxon in the order *Picornavirales*.

## DISCUSSION

Although viruses in the order *Picornavirales* exhibit a wide variety of genome structures, the arrangement of SPs and NSPs is distinct for monopartite picornaviruses in each of the 8 families that can be divided into TypeI (5′-SPs-NSPs-3′) (*Iflaviridae*, *Picornaviridae*, *Polycipiviridae*, and *Secoviridae*) and Type II (5′-NSPs-SPs-3′) (*Caliciviridae*, *Dicistroviridae*, *Marnaviridae*, and *Solinviviridae*) (https://ictv.global/report). In this study, 2 iflaviruses, IsIV1 and IsIV2, identified in the same host species damselfly (*I. senegalensis*) displayed the genome organizations of TypeI and TypeII, respectively. Subsequently, *in silico* screening of genome arrangement for the publicly available picornaviruses discovered the 2 genome types co-existing in at least 1 viral family (*Iflaviridae*).

Previously, the reversed picornaviruses genome arrangement of SPs and NSPs has been sporadically demonstrated only for viruses in the family *Iflaviridae*. Based on the inverted genome organization (TypeII) and the clustered phylogenetic relationship, it is recently proposed that BcPLV and DcPLV can be established as a new genus with the provisional name of “Psylloidivirus” in the family *Iflaviridae* (16) or even represent a new family (13). However, the reconstructed phylogenetic tree including those 10 identified iflaviruses with TypeII genome evidently illustrated that genome types (TypeI or TypeII) might not be the key factor for viral taxonomy in the family *Iflaviridae* (Fig. 4). Although the reversed genome arrangement of picornaviruses was currently not confirmed in the *Secoviridae* and *Dicistroviridae* families, our results suggested that there might be different viral classification criteria for the families within the order *Picornavirales*, even though genome organization is typically considered as a principal feature for the taxonomy of RNA viruses (4, 19).

Recombination is believed to be a major driving force for the diversity of RNA viruses. There are 2 forms of recombination for RNA viruses, including RNA recombination present in any type of RNA viruses (predominantly non-segmented RNA viruses, particularly with positive-sense RNA genome), and reassortment that is restricted merely to RNA viruses with segmented genomes (mainly during co-infection) (20, 21). However, the mechanism of SPs and NSPs exchange in picornaviruses discovered in *Iflaviridae* family might not be simply explained by either one of the above recombination types due to the swap of the entire regions of SPs and NSPs (Fig. 3A). Moreover, it is widely acknowledged that segmented RNA viruses are evolved from unsegmented viral ancestors, but not vice versa (22, 23), thus it is also not possible that the exchange of SPs and NSPs of the monopartite picornaviruses is derived from segmented ancestors. Although the authentic mechanism of the reversed SPs and NSPs genome structure of picornaviruses in the same family is still unclear, a reasonable hypothesis proposed in this study is that the 2 genome types (TypeI and TypeII) of these viruses might have the same viral ancestors with circular genome. This hypothesis is primarily based on the recent report for the highly genomic diversity of chuviruses with various genome forms including unsegmented, bi-segmented, and the circular form (23). Interestingly, the gene arrangement of circular chuviruses were mostly in the following order: the polymerase gene (L), the glycoprotein gene (G), and the nucleoprotein gene (N) (referred to as L-G-N), whereas the gene order of linear chuviruses can be L-G-N, or G-N-L, or N-L-G (32, and unpublished data). Therefore, it is assumed that the reversed genes of picornaviruses in the order *Picornavirales* might have underwent a similar evolutional process to chuviruses, and the circular form of picornaviruses was possibly eliminated or extinct during the evolution. Additionally, it is important to note that the foot-and-mouth disease virus (FMDV), another virus in *Picornaviridae* family, has been shown to have genome segmentation in cell culture, potentially serving as intermediates for block reversal and contributing to genome flexibility (24). However, further research is required to determine whether a similar scenario could explain the reversed genome structure in the picornaviruses examined in this study.

In summary, this study identified 2 iflaviruses with the inverted arrangement of SPs and NSPs in damselfly. Subsequent *in silico* systematic screening with all the currently publicly available picornaviruses led to the discovery of numerous picornaviruses with the non-canonical genome arrangement in *Iflaviridae* family, reflecting the potential prevalence of this phenomenon in the order *Picornavirales*. Nevertheless, with the fast-growing number of newly discovered viruses, particularly revealed by high-throughput sequencing, more and more complex genome diversity of picornaviruses will be demonstrated, which definitely contribute to a better understanding for the future taxonomy and evolution studies of viruses in the order *Picornavirales*.

## MATERIALS AND METHODS

### Sample preparation, RNA-Seq, and host insect identification.

Adult damselflies were collected alive with an insect net in August 2020 in Ningbo, China. The insects were then transferred to our laboratory and total RNAs were extracted from 2 adult damselflies using TRIzol reagent (Invitrogen) according to the manufacturer’s protocol. The extracted total RNAs were subsequently subdivided for transcriptome sequencing, small RNA (sRNA) sequencing, and Reverse Transcription-PCR (RT-PCR) verification, respectively. For transcriptome, a non-strand-specific cDNA library was created and RNA sequencing (paired-end 150 bp reads) was conducted on an Illumina HiSeq 4000 platform (Illumina). The raw data was processed with Trimmomatic (version 3.90) (25), and *de novo* assembly was performed using Trinity (version 2.8.5) with default parameters (26). The sRNA library was prepared using the Illumina TruSeq Small RNA Sample Preparation Kit (Illumina), and the sequencing was performed on an Illumina HiSeq 2500 by Novogene. To determine the accurate species of damselfly, a BLAST search was performed using the assembled contigs against the Barcode of Life Data Systems (https://www.boldsystems.org) and National Center for Biotechnology Information (NCBI) nucleotide (NT) database to identify the cytochrome oxidase subunit 1 (COI) sequence of the insect.

### Viral genome identification and confirmation.

To identify the potential viruses in the damselfly, the assembled contigs of the insect were searched (DIAMOND BLASTX) against a local virus database comprised of the NCBI viral reference database (https://www.ncbi.nlm.nih.gov/genome/viruses) (27). To avoid false positives, the discovered viral-like contigs were further searched against the online NCBI NT and non-redundant (NR) protein databases. Moreover, reverse transcription-PCR (RT-PCR) was performed to confirm the identified viral sequences, and then the viral genome termini were obtained by rapid amplification of cDNA ends (RACE) using the SMARTer RACE 5′/3′ kit (TaKaRa) followed by Sanger sequencing. Primers with sufficient overlap between adjacent products were designed for the identified viral sequences as listed in Table S1.

### Analysis of virus-derived sRNA.

First, adapter, low-quality, and junk sequences were removed from the raw reads of sequenced sRNAs, as described previously (28). Afterward, by using Bowtie software, sRNAs with a length of 18–30 nt were fetched and mapped into the genome sequences of the identified viruses (allowing zero mismatches) (29). The virus-derived sRNAs (vsiRNAs) were further extracted for downstream analysis (such as vsiRNA size preference, and distribution across the viral genome etc.) with Linux bash scripts.

### Retrieval of all publicly available picornaviruses from the eight families of the order *Picornavirales*.

To obtain all the publicly available picornaviruses, first, representative viral genome sequences of the 8 families (*Iflaviridae*, *Picornaviridae*, *Polycipiviridae*, *Secoviridae*, *Caliciviridae*, *Dicistroviridae*, *Marnaviridae*, and *Solinviviridae*) in the order *Picornavirales* were downloaded from the reference genome of NCBI (https://www.ncbi.nlm.nih.gov/data-hub/genome/) (retrieved on August 2022), respectively. Second, deduced amino acids corresponding to the RdRP domain of these picornaviruses were obtained for viruses in each family and used as the query searching (BLASTP) against NCBI NR database, so as to fetch all the currently available picornaviruses. Thereafter, the homologous picornaviruses were determined upon the E-value cutoff of 1 × 10^−5^ based on the blast results. Genome sequences of these picornaviruses with a genome length greater than 3000 bp were subsequently acquired. All the obtained genome sequences were then treated with the CD-HIT program (98% sequence identity threshold) (30) to produce a set of non-redundant representative viral sequences.

### *In silico* systematic screening to identify candidate picornaviruses with inverted genome organization of the families.

The conserved domains of candidate picornaviruses were determined with NCBI CD-Search tools (https://www.ncbi.nlm.nih.gov/Structure/bwrpsb/bwrpsb.cgi) and EBI InterProScan (https://www.ebi.ac.uk/interpro), and genome structures of these picornaviruses were then visualized by TBtools (31). Thereafter, picornaviruses with inverted genome organization of corresponding families were selected for further phylogenetic analysis.

### Phylogenetic analysis.

Phylogenetic trees were constructed using amino acid sequences of the predicted conserved RdRp or coat protein (CP) region of the selected picornaviruses, as well as corresponding regions of representative viruses in various families of the order *Picornavirales*, respectively. The best substitution model was evaluated and chosen using ModelTest-NG (32), and the trees were constructed with the Maximum likelihood (ML) algorithm by RAxMLNG (version 0.9.0) (1000 bootstrap replicates) (33). Based on the phylogenetic analysis results, the selected picornaviruses with inappropriate families were removed and the trees were reconstructed as described above.

### Data availability.

The raw sequence reads of damselfly transcriptome have been deposited in the SRA databases under accession SRR22252042. Genome sequences of the identified 2 iflaviruses in damselfly were deposited in the GenBank database under accession OP548101 and OP548102.
