# Supplementary material for: Prevalence of Reversed Genome Organizations for Viruses in the Family Iflaviridae, Order Picornavirales
Source: Microbiol Spectr. 2023 May 1;11(3):e04738-22. doi: 10.1128/spectrum.04738-22 (PMC10269833; doi:10.1128/spectrum.04738-22)

**Supplementary Table S1.** Primers used in this study.

**Supplementary File S1.** Genome sequences of Ischnura senegalensis iflavirus 1 and Ischnura senegalensis iflavirus 2.

**Supplementary File S2.** Genome sequences of all retrieved publicly available picornaviruses in eight families of the viral order *picornavirales*.

**Supplementary File S3.** Predicted genome structure and conserved domains for all of the publicly retrieved picornaviruses in eight families of the viral order *picornavirales*.

## Supplementary Table S1. Primers used in this study

| Primer | Sequence (5' to 3') | Purpose |
|---|---|---|
| Long primer | CTAATACGACTCACTATAGGGCAAGC AGTGGTATCAACGCAGAGT | Amplification of 5′/3′ RACE fragment |
| Short primer | CTAATACGACTCACTATAGGGC | |
| # IsIV2-5′-RACE GSP | CCCACTCCTCTATGATGCCTCAACTC CAC | Amplification of 5′ RACE fragment of IsIV2# |
| IsIV2-3′-RACE GSP | TGGCGTTGAAGATAGAATGAATGGG GTTA | Amplification of 3′ RACE fragment of IsIV2 |
| # IsIV1-3′-RACE GSP | TTCTTTTTATATGATATTATCCTTTGG TG | Amplification of 3′ RACE fragment of IsIV1# |
| IsIV2_1-F | TACCTGAGGAGCATGGACGA | IsIV2 viral genome:444-2887 |
| IsIV2_1-R | GGCGTATTCCGCTATCACCA | |
| IsIV2_2-F | TTAGGCGGATGGCATGTGTT | IsIV2 viral genome:2360-4824 |
| IsIV2_2-R | TGCTACGCCCAAAAACAACG | |
| IsIV2_3-F | ATGACTTTGCCGAGTCGTGT | IsIV2 viral genome:4228-6718 |
| IsIV2_3-R | GTACGGCAGTAGCATACCCC | |
| IsIV2_4-F | AGAGCGTGTAGTTTTGGCGA | IsIV2 viral genome:6342-8704 |
| IsIV2_4-R | GTGCCACATGATCTGACCCA | |
| IsIV2_5-F | CACAGCCTAGTATTGCCCCG | IsIV2 viral genome:8536-9037 |
| IsIV2_5-R | TCTTCAACGCCATCCAAGTGA | |
| IsIV2_6-F | GGTTTGCCCTGCTAACAAGA | IsIV2 viral genome:97-605 |
| IsIV2_6-R | TTGCCACTCAGCTACTACCAC | |
| IsIV1_1-F | GCTTAGCGGCCGAGATAGTT | IsIV1 viral genome:44-2934 |
| IsIV1_1-R | GAAGGCTTGAGCCAGAGTGT | |
| IsIV1_2-F | AGACTACTCATCGCCAGGGT | IsIV1 viral genome:2049-4837 |
| IsIV1_2-R | TGTTGCAGCAGCCGTAGTTA | |
| IsIV1_3-F | TGCGGTTGGTTCACTCCTAC | IsIV1 viral genome:4476-7400 |
| IsIV1_3-R | AAAGGGGTTTCATAGCGGGG | |
| IsIV1_4-F | GCTGGCGTATAAGCGTAGGT | IsIV1 viral genome:6454-8695 |

| | | |
|---|---|---|
| IsIV1_4-R | GTTGTGCAAGAAAGGGGCTG | |
| IsIV1_5-F | GCTGTTTTGTGGAGCACTGG | IsIV1 viral genome:7555-10111 |
| IsIV1_5-R | TTACTCGGCGAAGCACTGTT | |

# IsIV1: Ischnura senegalensis iflavirus 1

# IsIV2: Ischnura senegalensis iflavirus 2

# Virus genome organization in the family *Caliciviridae*

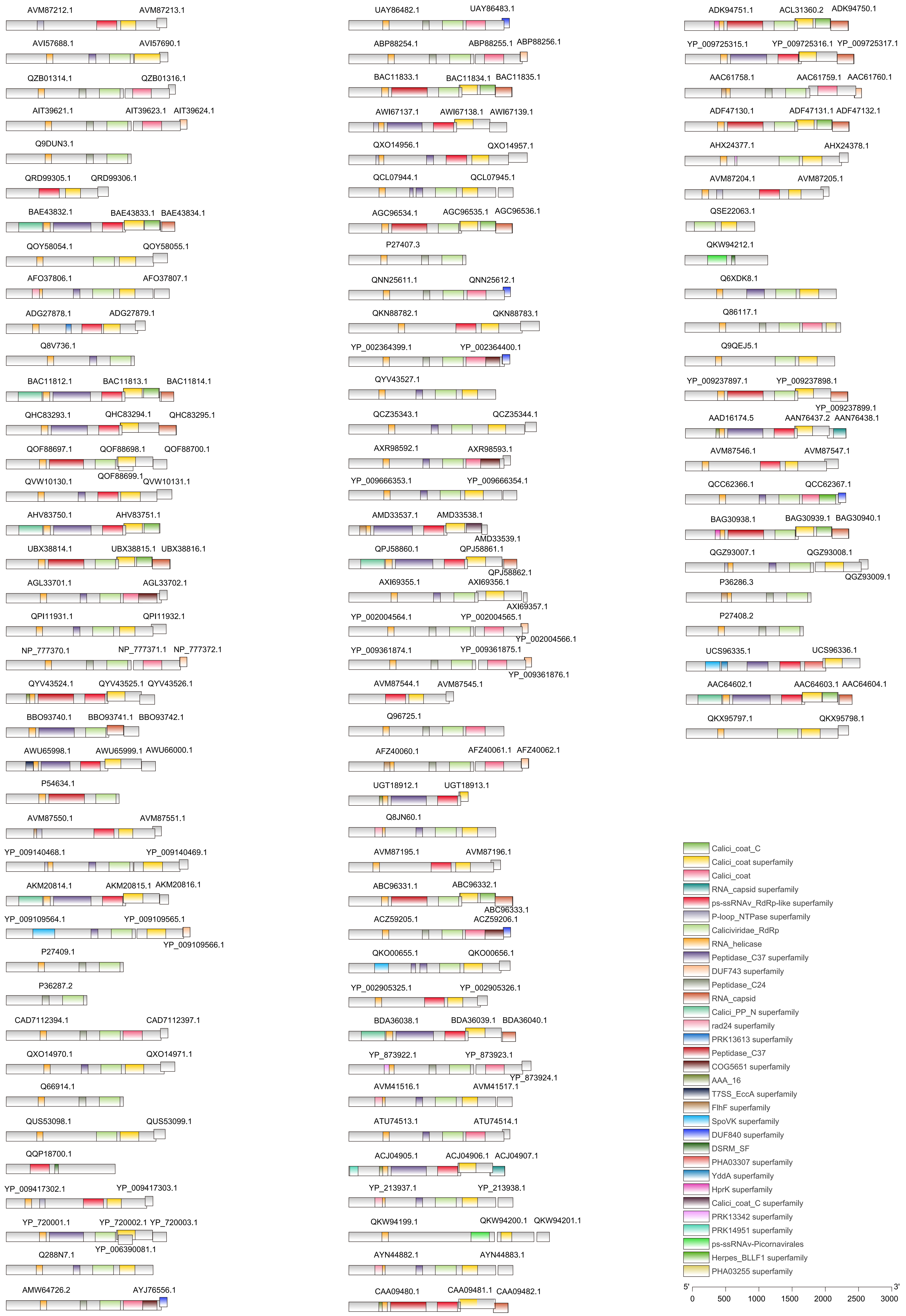

# Virus genome organization in the family *Dicistroviridae*

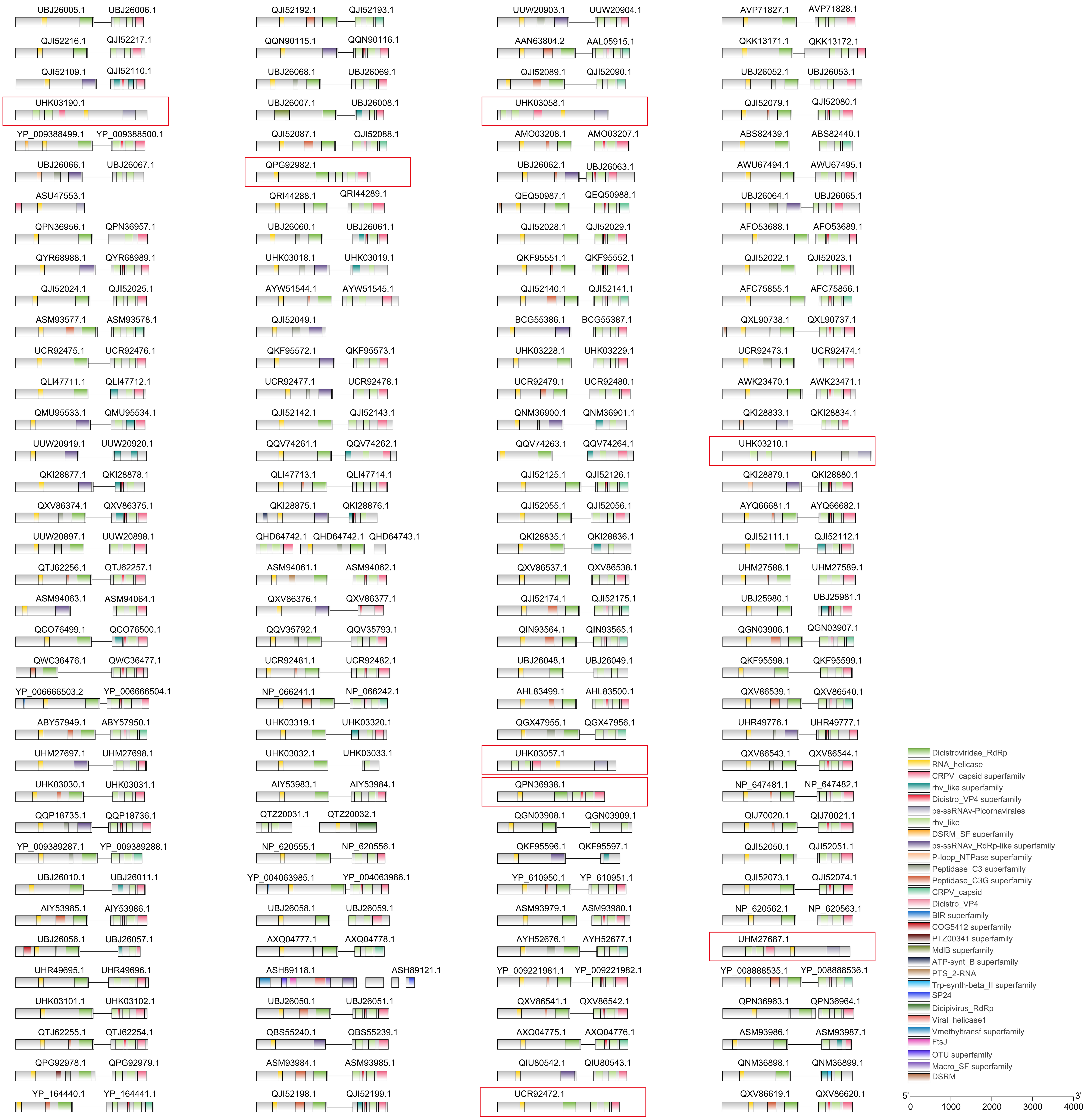

**Virus genome organization in the family *iflaviridae***

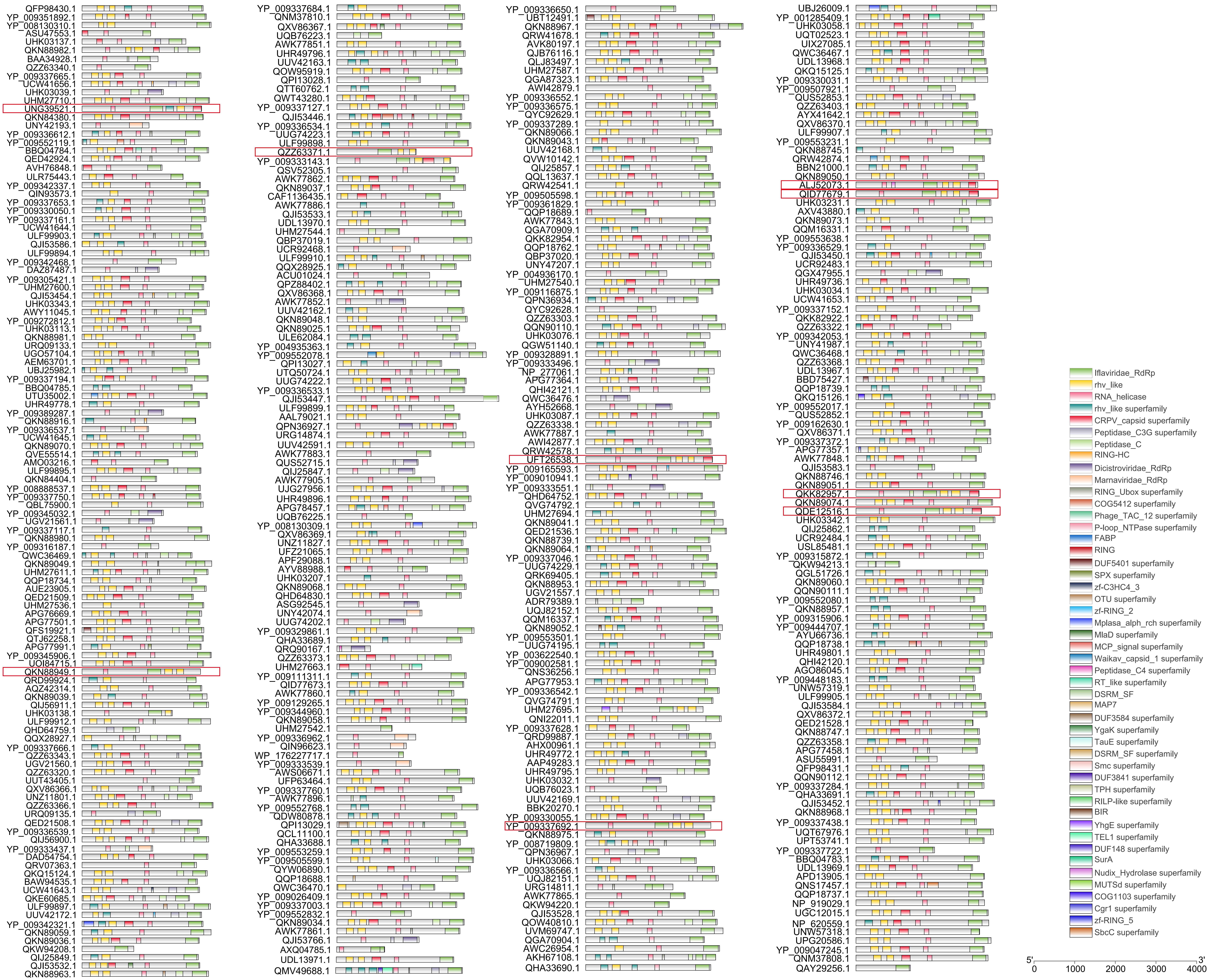

# Virus genome organization in the family *Marnaviridae*

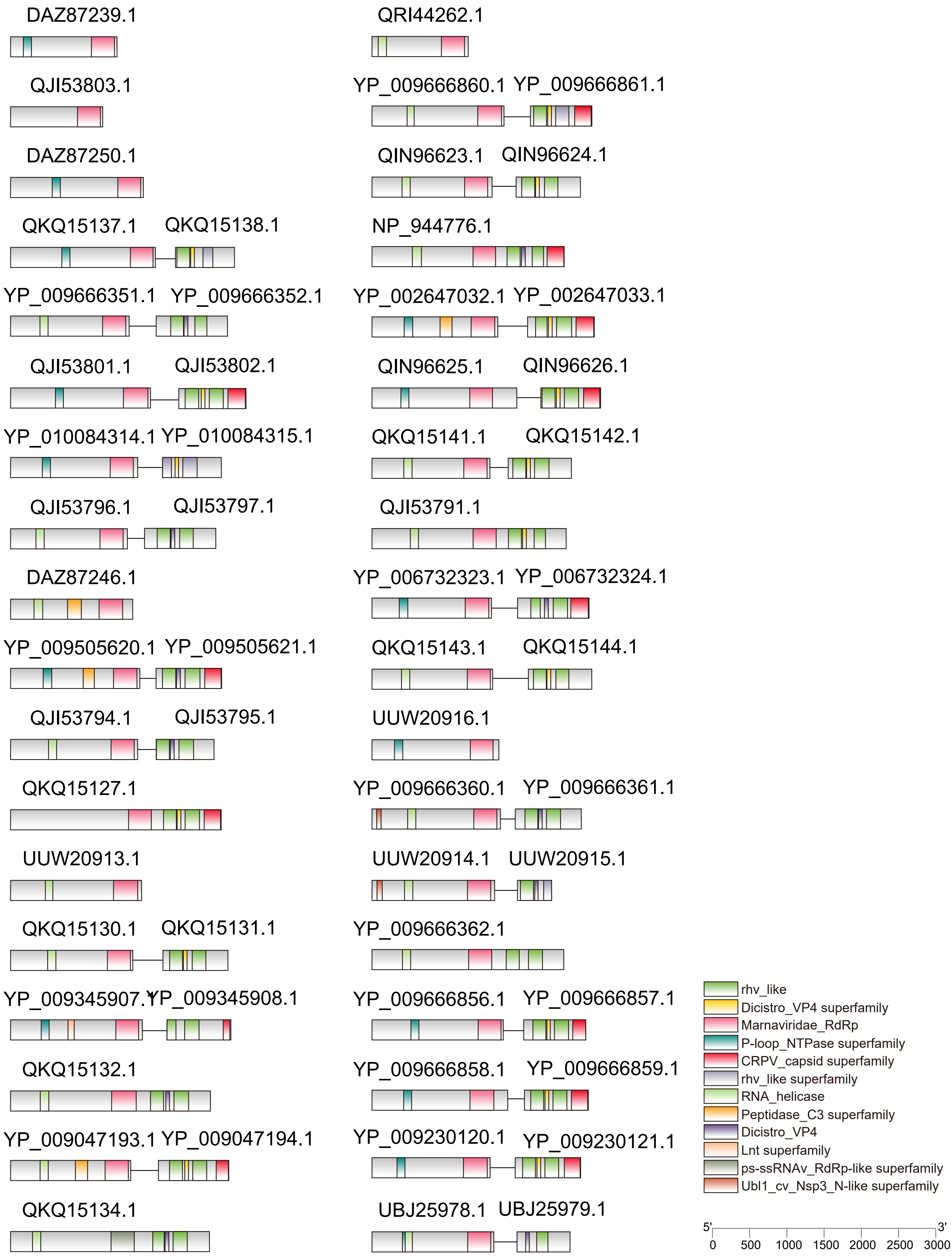

# Virus genome organization in the family *Picornaviridae*

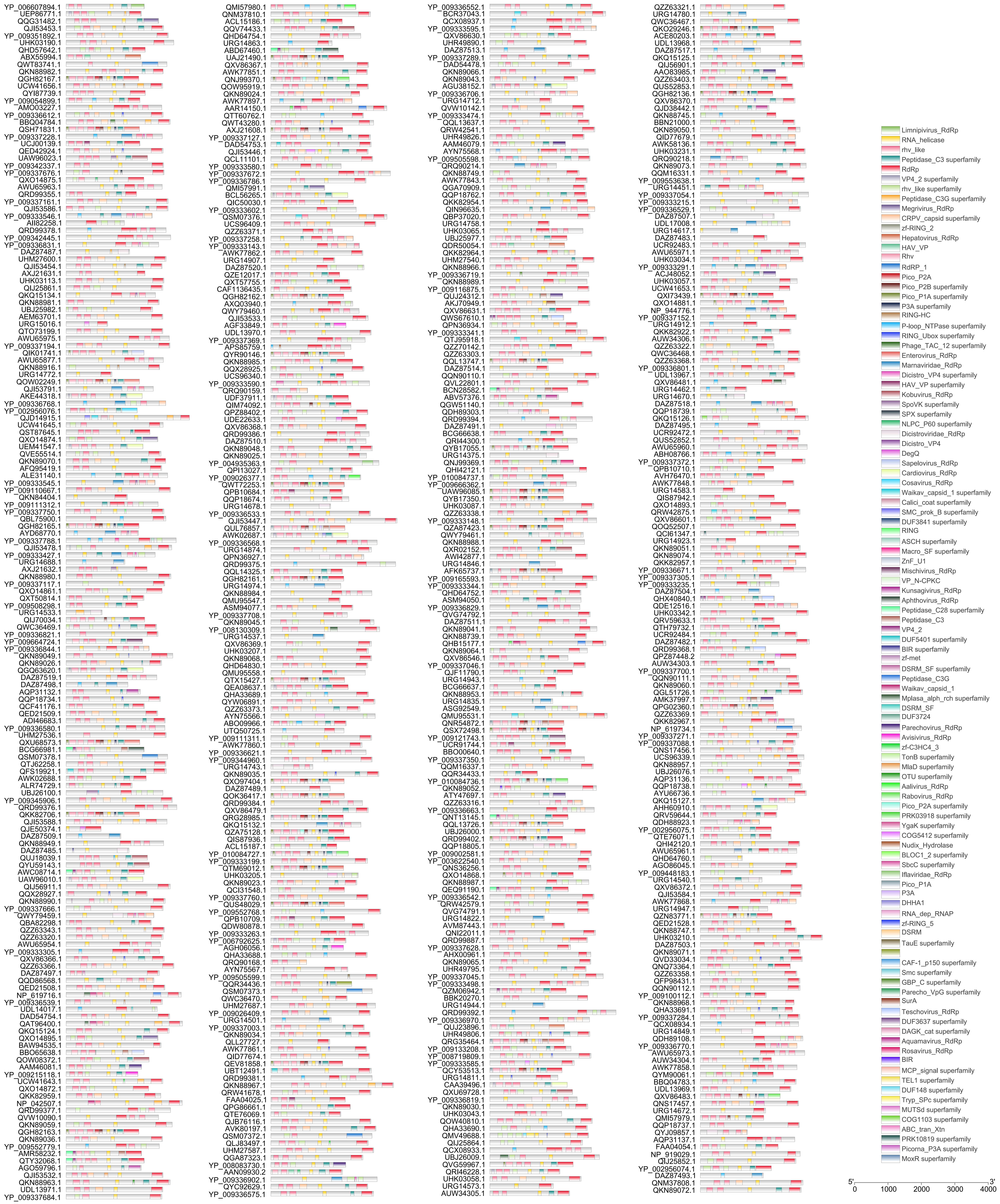

# Virus genome organization in the family *Polycipiviridae*

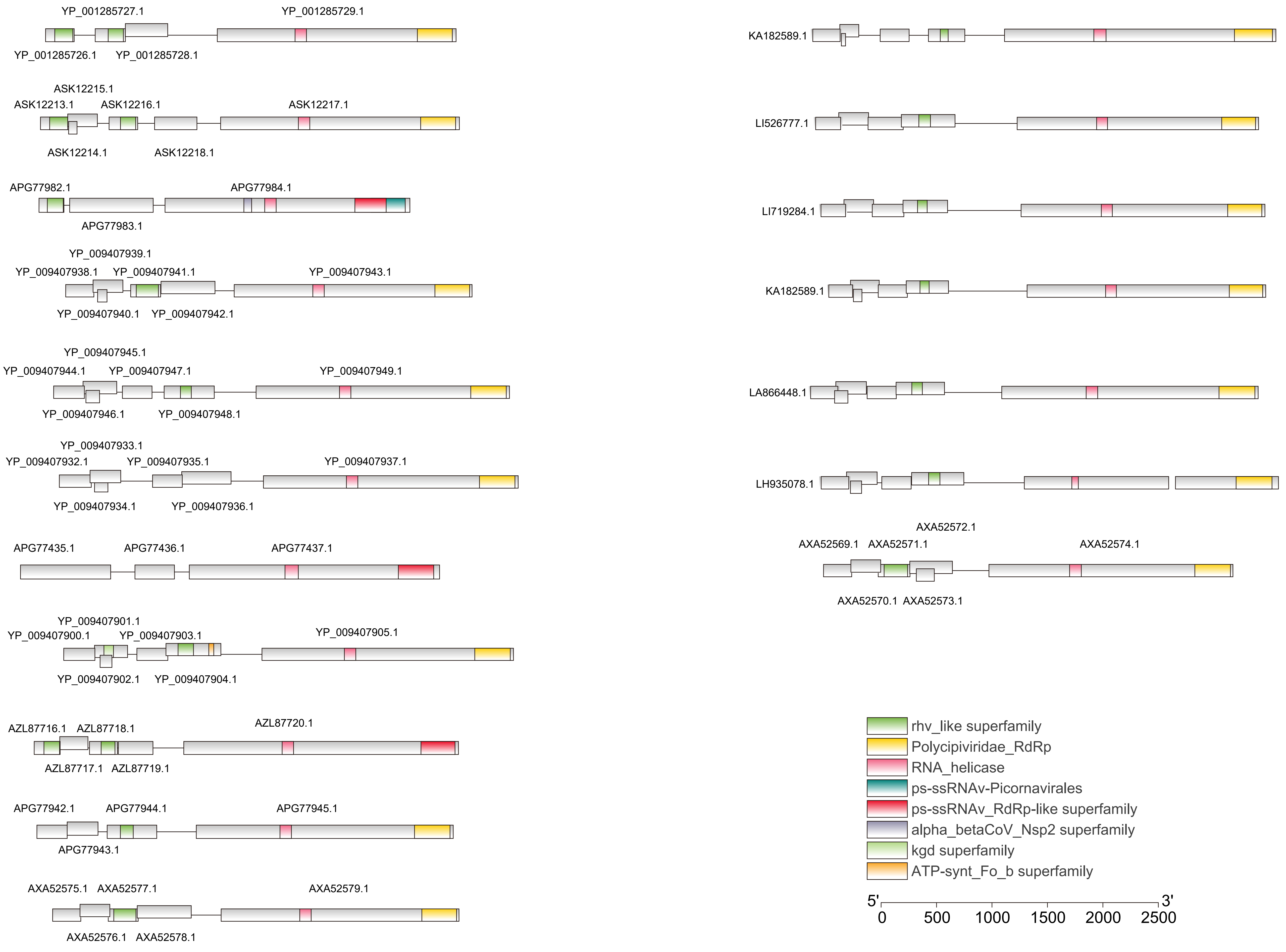

# Virus genome organization in the family *Secoviridae*

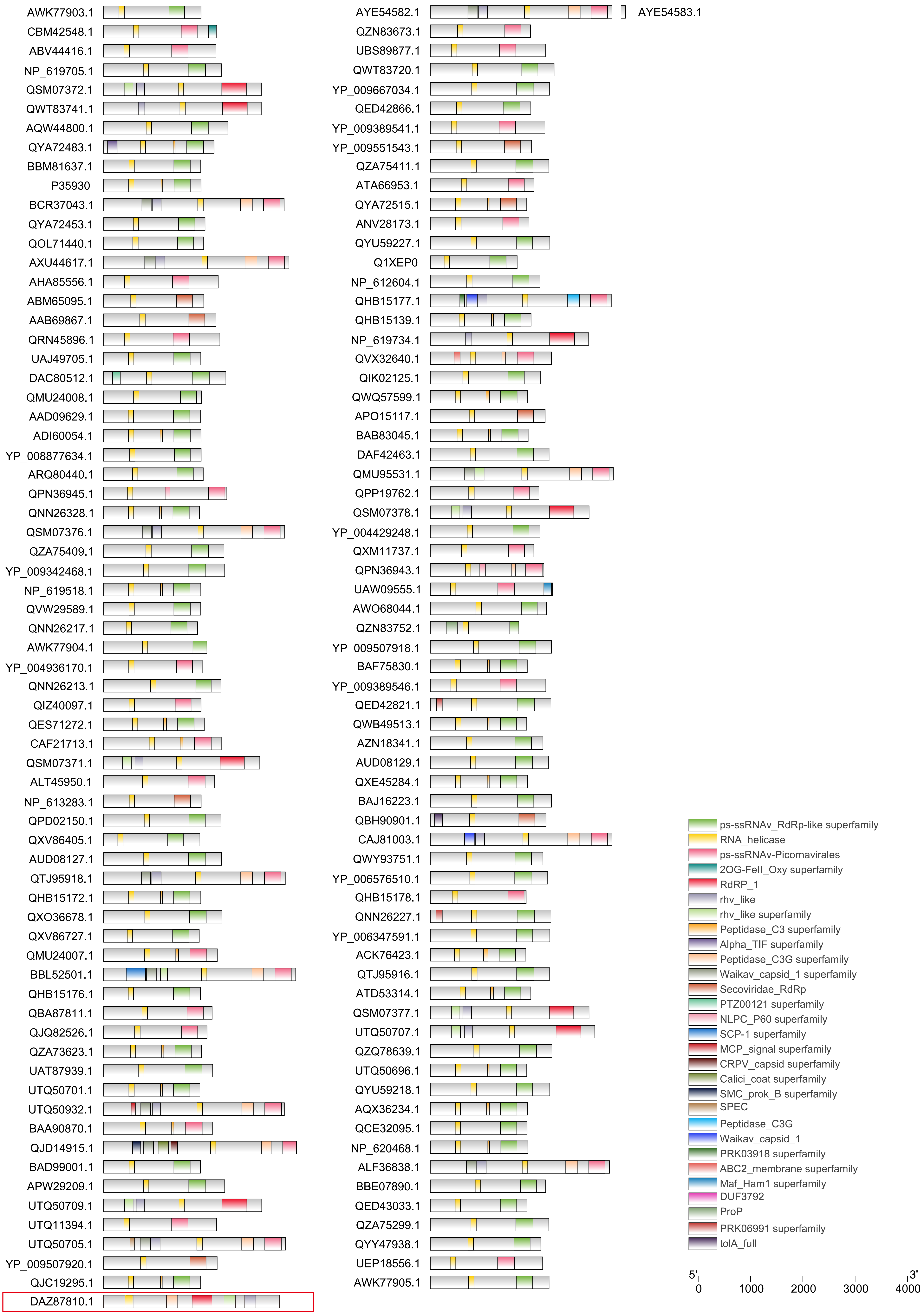

# Virus genome organization in the family *Solinviviridae*

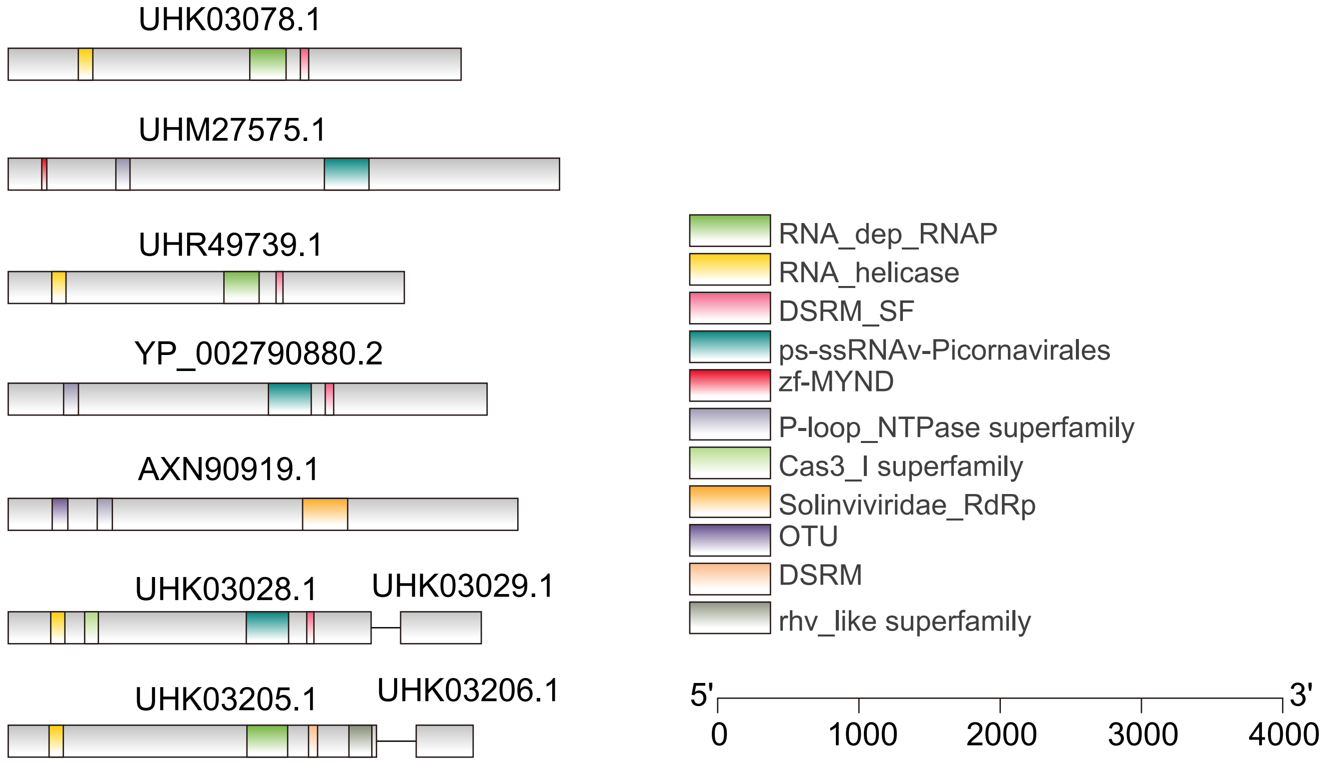

Supplement: Supplemental file 3 — Supplemental material. Download spectrum.04738-22-s0003.pdf, PDF file, 3.6 MB [file spectrum.04738-22-s0003.pdf]
